# Atherosclerosis Alters Loading-Induced Arterial Damage: Implications for Robotic Surgery

**DOI:** 10.1371/journal.pone.0156936

**Published:** 2016-06-13

**Authors:** Rachel Geenens, Nele Famaey, Andy Gijbels, Silke Verhelle, Stefan Vinckier, Jos Vander Sloten, Paul Herijgers

**Affiliations:** 1 Department of Cardiovascular Sciences, KU Leuven, Research Unit Experimental Cardiac Surgery, Herestraat 49 –Box 7003, 3000 Leuven, Belgium; 2 Department of Mechanical Engineering, KU Leuven, Celestijnenlaan 300 –Box 2419, BE-3001 Heverlee, Belgium; 3 Department of Oncology–Vesalius Research Centre, KU Leuven–VIB, Herestraat 49 –Box 912, 3000 Leuven, Belgium; INSERM, FRANCE

## Abstract

**Background:**

Lack of intra-operative haptic information during robotic surgery increases the risk for unintended tissue overload and damage. Knowledge about the acute and chronic fundamental relationship between force load and induced damage in healthy and diseased arteries is crucial to enable intra-operative haptic feedback or shared autonomy and improve patient safety.

**Methods:**

Arteries of wildtype and atherosclerotic mice were clamped *in vivo* for 2 minutes (0.0N, 0.6N or 1.27N). Histological analysis (Verhoeff’s-Van Gieson, Osteopontin, CD45, CD105) was performed immediately, or after 6 hours, 2 weeks or 1 month. Endothelium-dependent and–independent vasodilatation was assessed immediately or 1 month after clamping.

**Results:**

Endothelium dependent vasodilatation is worse after clamping of wildtype arteries, but is restored after one month. Clamping also results in flattening of the innermost elastic membrane of both genotypes, which is reversed over time for wildtype arteries but not for vessels from atherosclerotic mice. Higher osteopontin content in wildtype and *LDLR-/-* mice after 2 weeks suggests a phenotypic switch of the medial smooth muscle cells (SMCs), an effect that is reversed after 1 month. While inflammation in the intima diminishes, medial CD45 content rises through time in both genotypes. CD105 staining shows that even manipulation without clamping results in endothelial cell loss in both *LDLR+/+* and *LDLR-/-* mice.

**Conclusions:**

Arterial clamping induces different acute and long-term injury to the vessel wall of atherosclerotic and healthy arteries.

## 1. Introduction

The last decades, researchers and clinicians are developing new surgical instrumentation including robot-assisted devices, and are implementing minimally invasive techniques to improve patient safety and satisfaction [1]. Minimally invasive surgery (MIS) is increasingly used because it limits invasiveness and tissue damage. However, surgeon feedback regarding the interaction forces between surgical instruments and tissue is worse or even completely absent during robotic minimally invasive surgery (RMIS) [2]. This increases the risk of undetected tissue overload that can inflict collateral damage on microscopic and even macroscopic level [3]. Tissue outside the visual field is at highest risk of accidental mechanical overload.

Therefore, it is important to determine safety thresholds above which the induced damage is unacceptable (defined as permanent and/or having irreversible consequences). Implementing these safety thresholds in robotic instruments with shared autonomy to avoid tissue overload has the potential to significantly increase surgical safety using robotic systems. Defining these thresholds poses a challenge, since it requires knowledge on the quantitative relation between mechanical loading and tissue damage. In our domain, intentional clamping as well as accidentally compressing arteries by instruments are occasionally occurring conditions, on which we focused.

The effects of different vascular clamps and ligatures on blood vessels have been studied by several research groups. It was shown histologically and functionally that higher clamping or compression forces induce higher tissue damage [3–8] and that the extent of damage is partly time-dependent [9]. Nevertheless, a defined quantitative relation between clamping load and induced damage for mouse thoracic aortas is still lacking. Furthermore, few studies focus on the long term effects of arterial clamping [10–12]. Since we define unacceptable damage as irreversible, we aim to study the intermediate term effects of well-defined clamping loads in surviving subjects. This enables us to investigate whether or not acute clamping-induced damage can be resolved.

At present, it is also insufficiently known whether these safety thresholds are influenced by age or pathology, in other words, whether they are patient-specific. Therefore we aim at studying the effect of age and atherosclerosis on load-induced aortic damage in well-controlled experimental mouse models, well known by our group. We have previously shown that clamping thoracic mouse aortas up to 2.0N (6 times the minimal occlusion force) [3] induces damage in C57BL/6J mice of 10, 25 and 40 weeks of age, without important differences in vascular wall response between these age groups [13].

Since a large proportion of patients undergoing surgery suffer from atherosclerosis, in this study we investigated an *LDLR* knock-out mouse model with the same C57BL/6J genetic background, that develops atherosclerotic lesions throughout the arterial tree when fed a western diet [14].

Acute and long-term effects of *in vivo* arterial clamping were quantified in wildtype and atherosclerotic mice. Clamps were not positioned on mature lesions. Our results could be of clinical importance to ensure proper patient-specific safety during robotic MIS.

## 2. Material and Methods

### 2.1. Animals

All animal experiments were approved by the ethical committee of KU Leuven (project P009/2011). Mice were housed in a 12-hour light/dark cycle and were fed at libitum. Only male animals were used to avoid inter-gender differences. C57BL/6J mice knockout for the *LDLR* were used, which were fed a western diet (Ssniff® EF R/M acc. TD88137 mod.) for 15 weeks, starting from 5 weeks of age [14]. All animals were genotyped to assure homozygous knockout of the *LDLR* (forward: 5'-CCATATGCATCCCCAGTCTT-3', reverse wildtype: 5'-GCGATGGATACACTCACTGC-3’, reverse knockout: 5’-AATCCATCTTGTTCAATGGCCGATC-3'). As wildtype control animals we used *LDLR+/+* mice, fed standard chow for 20 weeks.

### 2.2. Surgical procedure

Different animals were used for functional en histological tests. 108 *LDLR+/+* and 108 *LDLR-/-* mice (6 mice per condition, see Table 1 for an overview of the different experiments) of 20 weeks were anesthetized (intraperitoneally, medetomidine 0.5mg/kg, Orion Pharma and ketamine 50mg/kg, Eurovet), intubated and ventilated (250μl stroke volume, 140 strokes/min). Rectal temperature was kept at 37°C.

**Table 1:** Overview of the experiments that were conducted in C57BL/6J *LDLR+/+* and *LDLR-/-* mice.

	Acute		6 hours	2 weeks	1 month	
	Histology	Function	Histology	Histology	Histology	Function
**0.0N**	n = 6	n = 6	n = 6	n = 6	n = 6	n = 6
**0.6N**	n = 6	n = 6	n = 6	n = 6	n = 6	n = 6
**1.27N**	n = 6	n = 6	n = 6	n = 6	n = 6	n = 6

Through a left thoracotomy, the descending thoracic aorta was carefully isolated from surrounding tissue and either used as a control sample (unclamped, 0.0N) or clamped *in vivo* for 2 minutes with a microvascular clamp at 0.6N (FST, 18055–01) or 1.27N (FST, 18055–02) (Table 1). The clamping jaws (2mm wide) are not serrated to allow homogenous distribution of the clamping forces. Afterwards, the clamp was removed and segments of the aorta were either immediately excised or the chest was closed. The mice were closely observed individually during recovery in a recovery chamber, where extra oxygen was supplied and body temperature was monitored, until they were fully awake. If an animal appeared weak, glucose was administered intraperitoneally, but we did not detect signs of pain requiring analgetics. In the survival study conditions, the aortic segment was excised 6 hours, 2 weeks or 1 month after initial clamping (Table 1, anesthesia as described above) after euthanasia by bleeding (cutting the heart). Excised segments were either tested for functional integrity or used for histological analysis.

### 2.3. Functional integrity testing

To test functional integrity, the aortic segments (1.68 ± 0.24 mm in length) were mounted in a water-jacketed organ chamber containing Krebs solution (Sigma-Aldrich, in mM: NaCl 118.3, KCl 4.7, KH_2_PO_4_ 1.2, MgSO_4_ 1.2, NaHCO_3_ 25, Na_2_Ca EDTA 0.026, Glucose 5.5, CaCl_2_ 2.5) at 37°C, aerated by 95% O_2_/ 5% CO_2_. Tension was measured isometrically with calibrated Type 375 force transducers (Hugo Sachs Elektronics) connected to a data acquisition system (NI-USB-6009, National Instruments). Segments were gradually stretched over 1 hour until a stable preload of 20 mN was reached. This preload was defined as optimal in preliminary tests, in which segment response to 40 mmol/l K^+^ was recorded (data not shown). After preload adjustment, preconstriction was induced by phenylephrine (PE, Sigma-Aldrich, 10^-6^M, 20 minutes). Subsequent cumulative addition of acetylcholine (ACh, Sigma-Aldrich, 10^-9^M to 10^-5^M) during 30 minutes allowed assessment of endothelium-dependent vasodilation, expressed as % reversal of PE-induced constriction. The segment was washed 3 times with fresh Krebs solution. Afterwards, endothelium-independent vasodilation was examined by adding a direct NO donor (sodium nitroprusside, SNP, Fluka, 10^-9^M to 10^-5^M) during 30 minutes following preconstriction by PE (10^-6^M, 20 minutes).

### 2.4. Histological analysis

Excised segments were fixated overnight in paraformaldehyde (4%), followed by dehydration (Medite TES 99) and embedding in paraffin. Serial cross-sections (7μm, 10 series) were made (Microm HM360). All stainings were examined using a Zeiss Axioplan 2 microscope and pictures were obtained with an Axiocam MRc5 camera.

#### Fluorescent immunohistology (CD105 and CD45)

Redundant details for these stainings are provided in Table 2.

**Table 2:** Protocol details for the fluorescent immunohistological stainings (CD105 and CD45).

	CD105	CD45
**Blocking serum**	Rabbit	Goat
** **	DAKO	DAKO
** **	1/5	1/5
**Primary**	Goat anti-CD105	Rat-anti-CD45
**antibody**	R&D AF 1320	BD Pharmingen
** **	1/50	1/100
**Secundary**	RaG-B	GaRat-B
**antibody**	DAKO	BD Pharmingen
** **	1/300	1/100
**Amplification**	TSA Cyanine3 System	TSA Cyanine3 System
** **	Perkin Elmer	Perkin Elmer
**Mounting**	ProLong® Gold Antifade	ProLong® Gold Antifade
**medium**	mountant with DAPI	mountant with DAPI
** **	Invitrogen	Invitrogen

**Fig 1:**
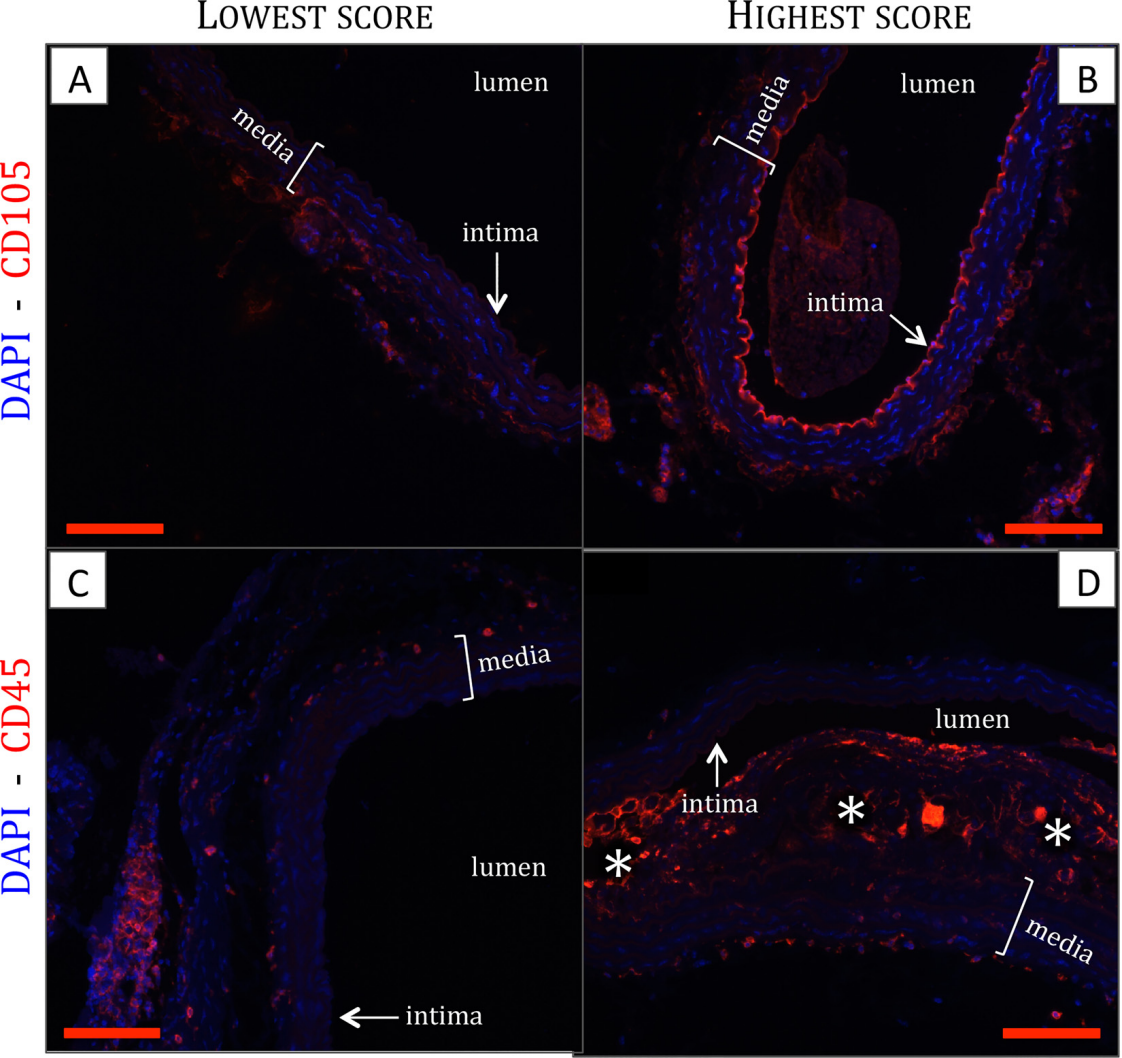
Representative images of CD105 (A,B) and CD45 (C,D) staining, showing the condition of the lowest score (A,C) and the highest score (B,D). *: atherosclerotic plaque, scale bar: 200μm.

**Fig 2:**
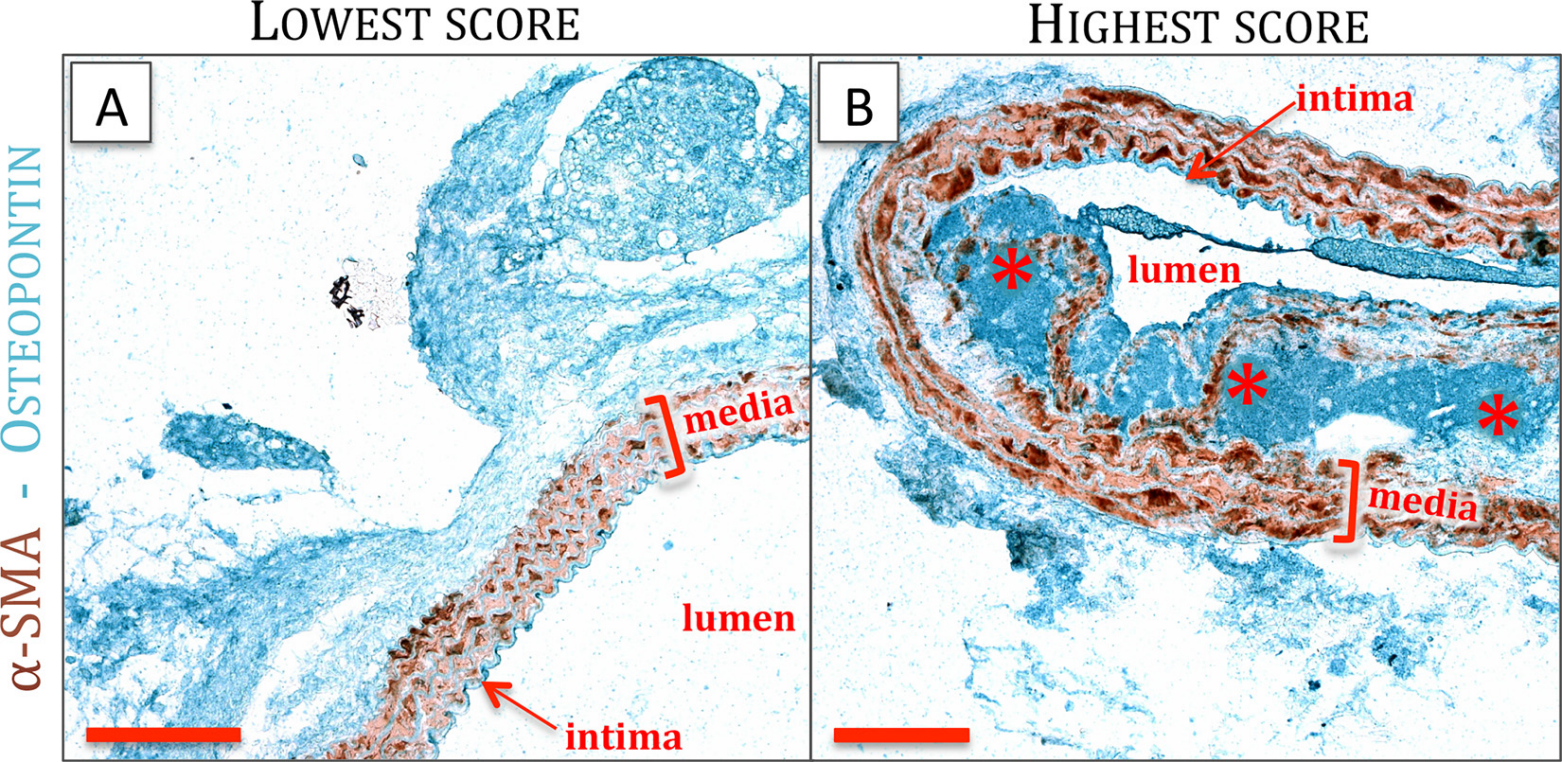
Representative images of osteopontin staining, showing the condition of the lowest score (A) and the highest score (B). *: atherosclerotic plaque, scale bar: 200μm.

**Fig 3:**
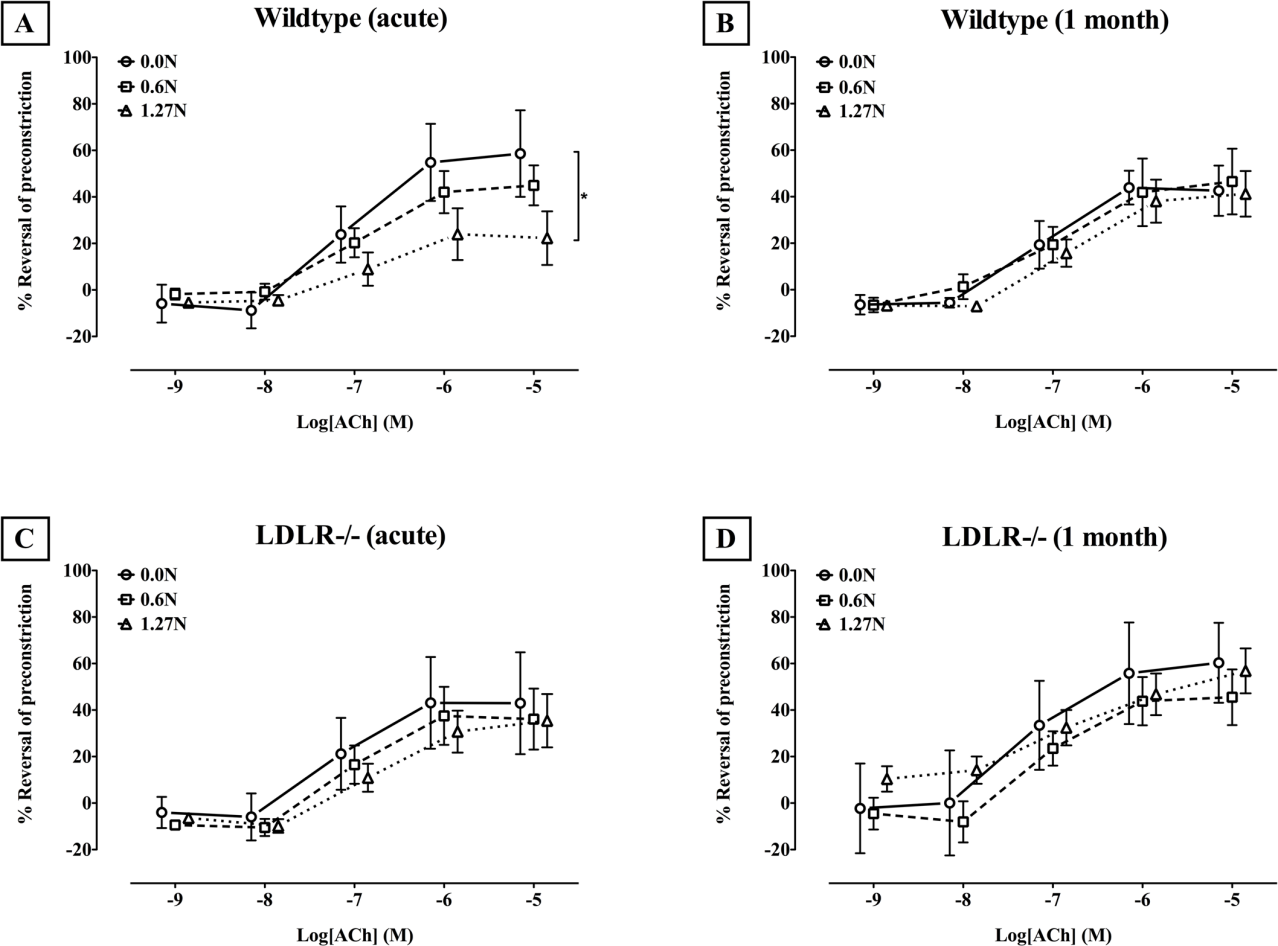
Relative endothelium-dependent relaxation due to cumulative ACh administration after PE-induced preconstriction (Mean ± SD). Dose-response curves are shown for wildtype (A,B) and LDLR-/- (C,D) mice, both for segments tested immediately after clamping (A,C) or after one month survival (B,D). * p<0.05.

**Fig 4:**
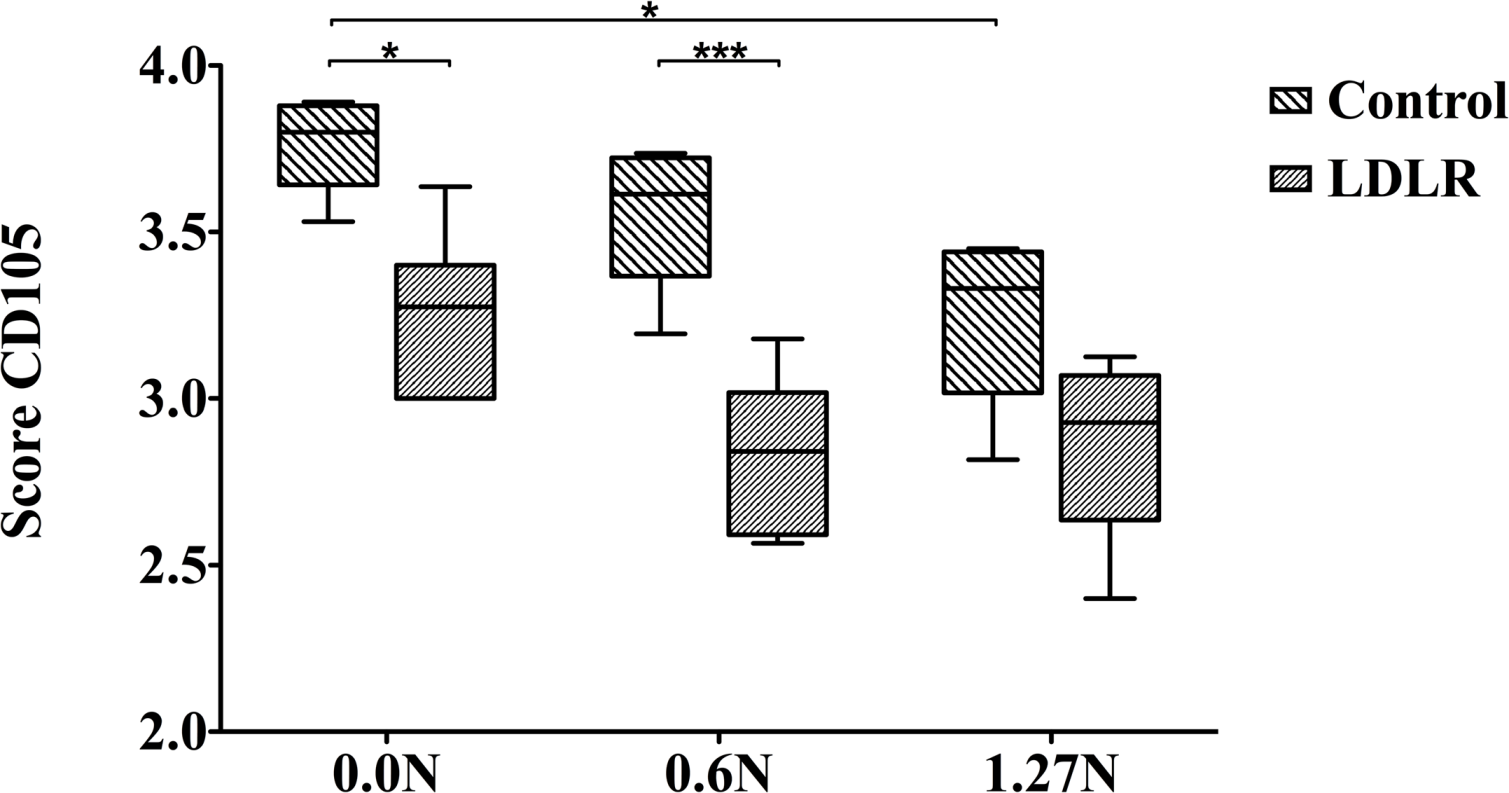
The effect of clamping on CD105 presence, displayed as percentage fluorescence detected in intima and media. Results of wildtype and *LDLR* knockout mice are shown immediately after clamping (Median and IQR, 5–95 percentile). * p<0.05, *** p<0.0001.

**Fig 5:**
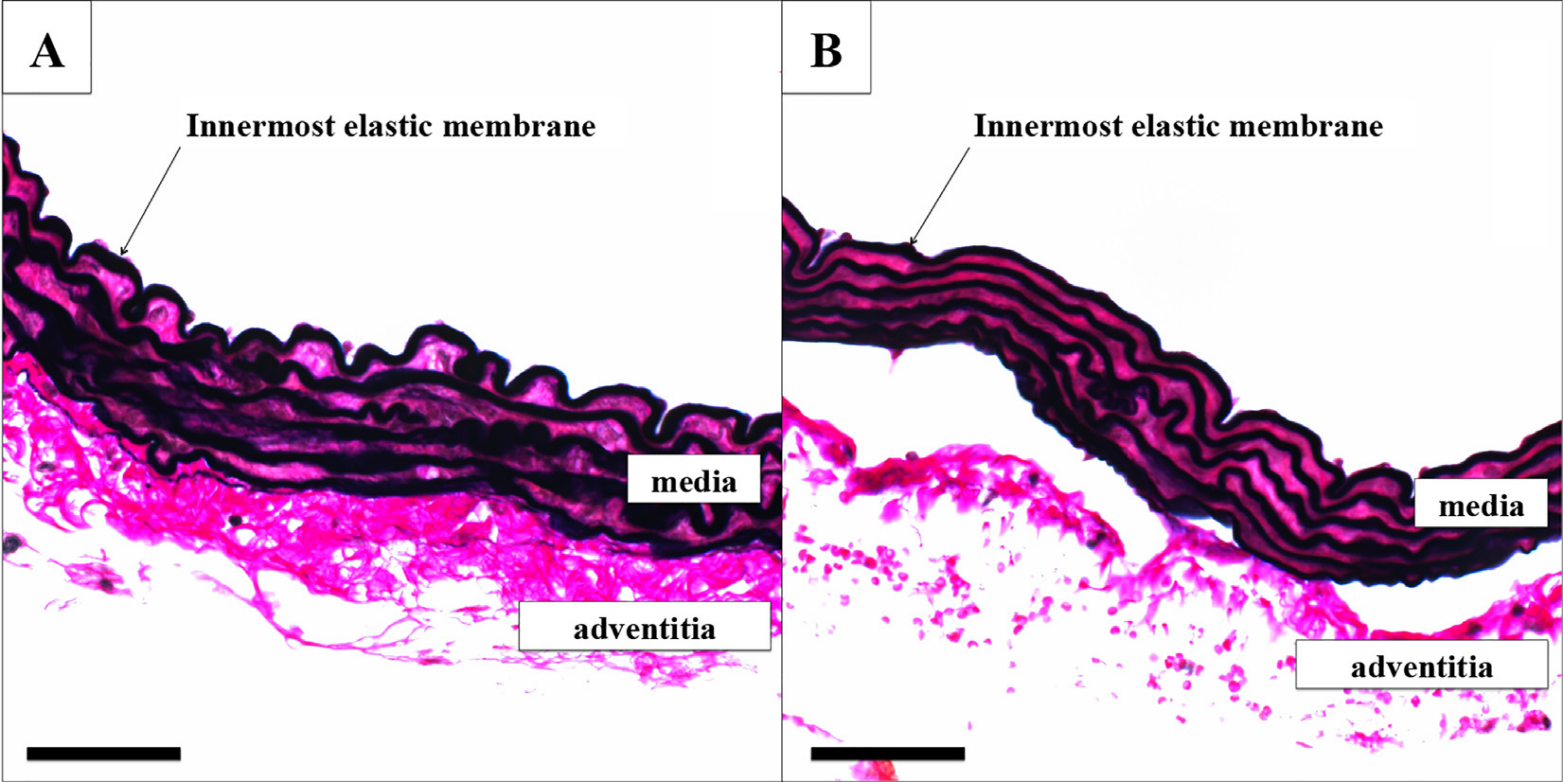
Verhoeff’s-Van Gieson staining of an unclamped (A) and 1.27N clamped (B) wildtype aorta. Scale bar 100μM.

**Fig 6:**
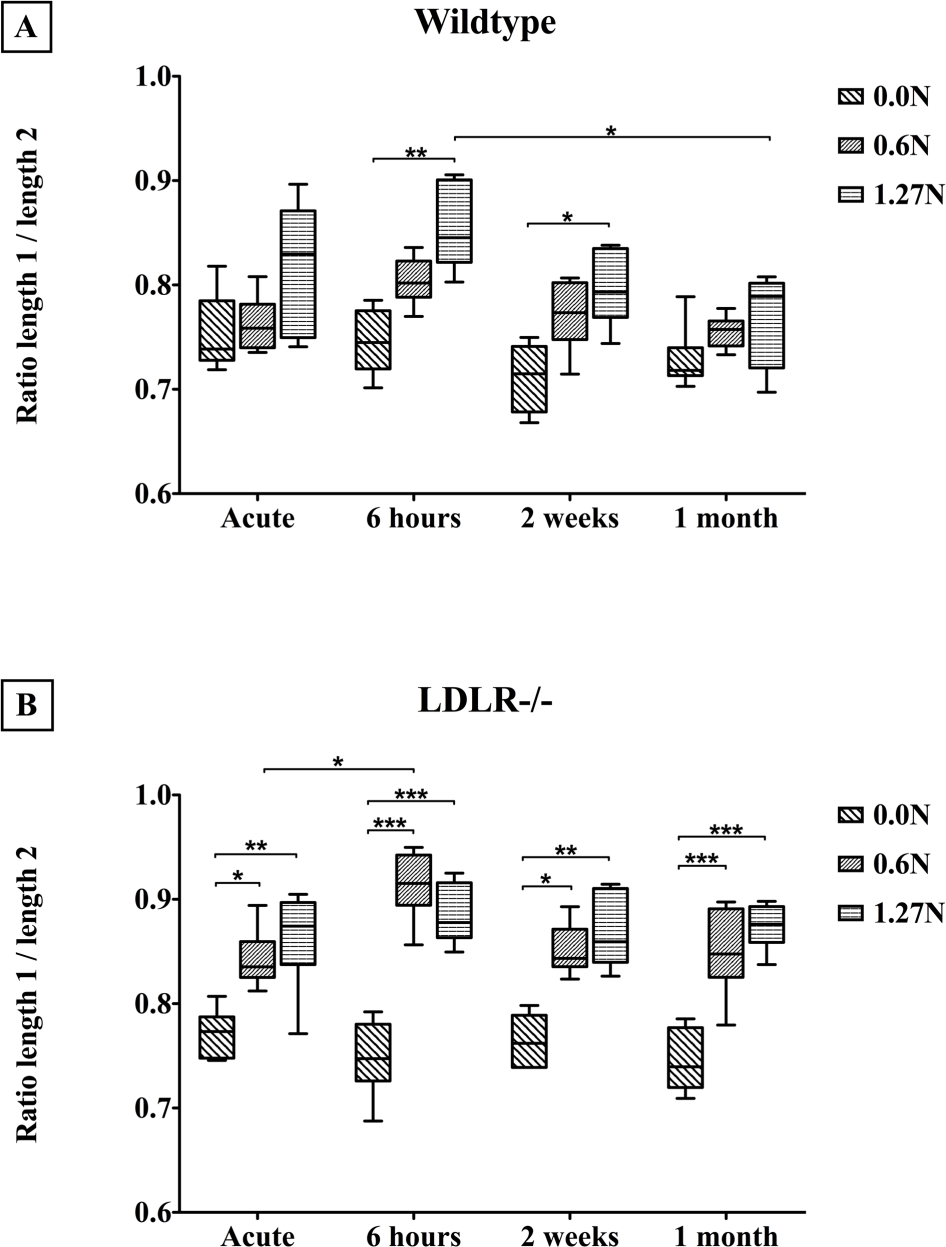
Longterm effects of clamping on the degree of flattening of the innermost elastic membrane for wildtype (A) and *LDLR-/-* mice (B) (Median and IQR, 5–95 percentile). Length 1: straight length, length 2: curved length. * p<0.05, ** p<0.001, *** p<0.0001.

In general, after hydration, antigen retrieval, block of endogenous peroxidases and block of nonspecific sites, primary antibody is incubated overnight. The next day, secondary antibody containing 10% mouse serum (Sigma) is applied, followed by amplification of the signal. Every section was divided in four quadrants, each of which was scored in a blinded manner, where the lowest score represents no fluorescence and the highest score maximal fluorescence (1 (Fig 1A) to 5 (Fig 1B) for CD105 and 1 (Fig 1C) to 6 (Fig 1D) for CD45). Quadrant scores were averaged and used for analysis.

#### Elastic membranes: Verhoeff’s-Van Gieson

After hydration, slides were immersed in fresh Verhoeff’s elastic staining solution (as described in [15]) for 22’, rinsed and immersed in 2% FeCl_3_ (2’15”), followed by rinsing and 2’ ethanol 95%. Then, slides were immersed in Van Gieson solution (Prosan) for 6’. After dehydration slides were mounted with DPX (Klinipath). The curved as well as straight-through length of the innermost elastic membrane were measured (blinded, ImageJ). The ratio (straight length divided by curved length) was used as a value for the amount of flattening: a value of 1 would mean a completely flat membrane.

#### Smooth muscle cell (SMC) phenotype: Osteopontin– α-SMA

Hydration was followed by antigen retrieval (Target Retrieval Solution, DAKO) and block of endogenous peroxidases. Application of goat serum (45’, 1/5, DAKO) blocks nonspecific sites. The first primary antibody was applied overnight (1/500, rabbit anti-α-SMA, Abcam ab5694) followed by incubation with Goat anti Rabbit IgG, conjugated with Horseradish peroxidase (45’, 1/100, DAKO P0448) containing 10% mouse serum (Sigma) and visualization by 3,3’-Diaminobenzidine (DAKO K346711). After thorough rinsing and block with donkey serum (45’, 1/5, Sigma), the second primary antibody is applied overnight (1/200, Goat anti-osteopontin, R&D AF808). The next day, incubation of the biotinylated secondary antibody (45’, 1/300, DAG-B, Santa Cruz SC2042) is followed by signal amplification (30’, Streptavidin-Alkaline Phosphatase, Abcam 64268) and visualization using green chromogen (Enzo ADI-950-160-1). Slides are mounted with Faramount (DAKO, S3025). Every section was divided in four quadrants, each of which was scored (1: no osteopontin, (Fig 2A) to 5: maximal osteopontin (Fig 2B)) for osteopontin presence. Quadrant scores were averaged in a blinded manner and used for analysis of SMC phenotype. Negative control stainings (no primary antibody, data not shown) were thoroughly studied before, to avoid taking blood remains into account in our scoring system.

### 2.5. Statistics

For isometric tension tests, preload was subtracted from the isometric tension value and all values were normalized for the length of the tested segment. Results are displayed as mean ± standard deviation. Statistical analyses (repeated measures two-way ANOVA) were performed with Statistica. For the histology results we aimed to analyze and pool at least 10 slices for every animal, and two way ANOVA was performed using SAS 9.4. P-values in the text represent results of the two way ANOVA testing. Posthoc p-values are displayed in the graphs and P < 0.05 was considered statistically significant.

## 3. Results

### 3.1. Endothelium-dependent and–independent vasodilatory function

Preconstriction induced by PE before ACh and SNP was comparable (p = 0.5309, data not shown). In the wildtype mice, we observed an acute deterioration of dose-dependent **endothelium-dependent vasodilation** after clamping (p = 0.0026, Fig 3A). While maximal relaxation due to 10^-5^M ACh additions was not impaired in arteries clamped with 0.6N (p = 0.3374), segments clamped with 1.27N showed a significantly lower response compared to the unclamped segments (p = 0.0130). One month after clamping, no difference in endothelium-dependent vasodilation was observed between clamped and unclamped arteries (p = 0.9964, Fig 3B). In *LDLR* knockout animals with atherosclerosis, clamping did not further deteriorate endothelium-dependent vasodilatation in the acute setting (p = 0.9778, Fig 3C) or after 1 month (p = 0.4135, Fig 3D). No difference between wildtype and *LDLR* knockout arteries could be observed for any of the ACh concentrations. Clamping did not impair acute or 1 month **endothelium-independent vasodilation** by SNP in wildtype nor in *LDLR* knockout samples (data not shown).

### 3.2. CD105: endothelial cells

Clamping results in lower CD105 content in the acute condition (p<0.0001, Fig 4). CD105 score decreases in time in both wildtype (p<0.0001) and atherosclerotic (p<0.0001) arteries, for unclamped (p<0.0001), 0.6N (p<0.0001) and 1.27N (p = 0.0025) clamped segments. In the acute situation, wildtype samples have higher CD105 score compared to *LDLR-/-* (p<0.0001, Fig 4).

### 3.3. Verhoeff’s-Van Gieson: elastic membranes

We did not detect rupture of the elastic membranes due to mechanical clamping at 0.6N nor at 1.27N. However, clamping induces flattening of the innermost elastic membrane, expressed by the ratio between curved and straight-through length, in wildtype (p<0.0001) as well as *LDLR-/-* (p<0.0001) arteries (Figs 5 and 6). Flattening is more pronounced in *LDLR-/-* compared to wildtype vessels at the acute (p = 0.0011), 6 hours (p = 0.0002), 2 weeks (p<0.0001) and 1 month (p<0.0001) time point. This is the case for unclamped (p = 0.0213), 0.6N clamped (p<0.0001) and 1.27N clamped (p<0.0001) segments. While in wildtype segments the clamping-induced flattening is reversed through time (p = 0.0006), this is not the case for atherosclerotic vessels (p = 0.0757) (Fig 6).

### 3.4. Osteopontin: smooth muscle cell phenotype

**Fig 7:**
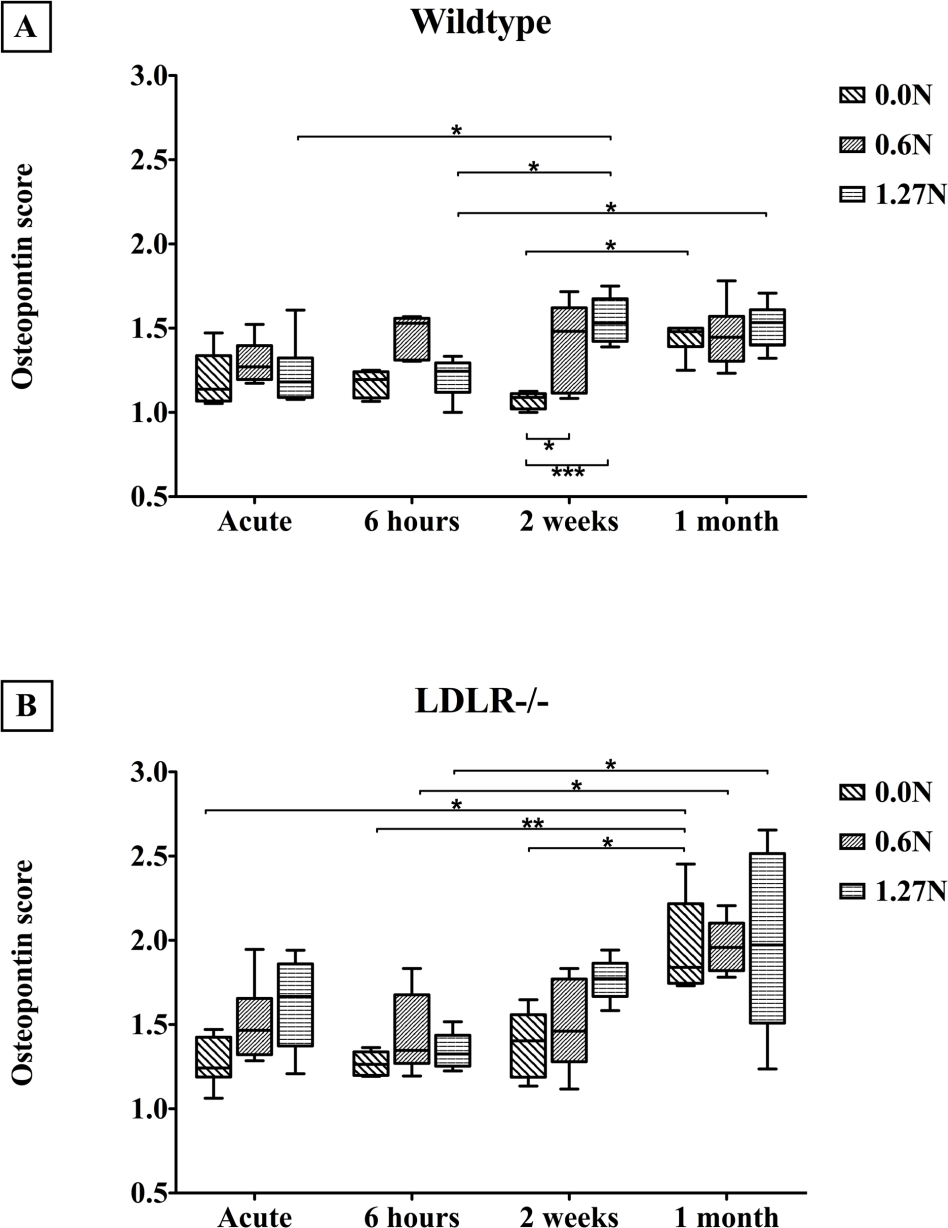
Osteopontin score in wildtype (above) and *LDLR-/-* (below) arteries on the different time points after clamping (Median and IQR, 5–95 percentile). * p<0.05, ** p<0.001.

α-SMA was also stained to improve interpretation. Osteopontin score is higher in *LDLR-/-* compared to wildtype arteries in unclamped (p<0.0001), 0.6N clamped (p = 0.0024) and 1.27N clamped (p = 0.0002) samples and is higher at later time points (p<0.0001; p = 0.0032 and p = 0.0002, respectively, Fig 7). Clamping results in higher osteopontin scores in wildtype (p = 0.0001) and *LDLR-/-* (p = 0.0256) arteries. Moreover, there was a significant effect of clamping in the 6 hours (p = 0.0009) and 2 weeks (p<0.0001) condition (Fig 7). This is not observed in the acute (p = 0.0560) and 1 month (p = 0.8969) condition.

### 3.5. CD45: inflammation

**Fig 8:**
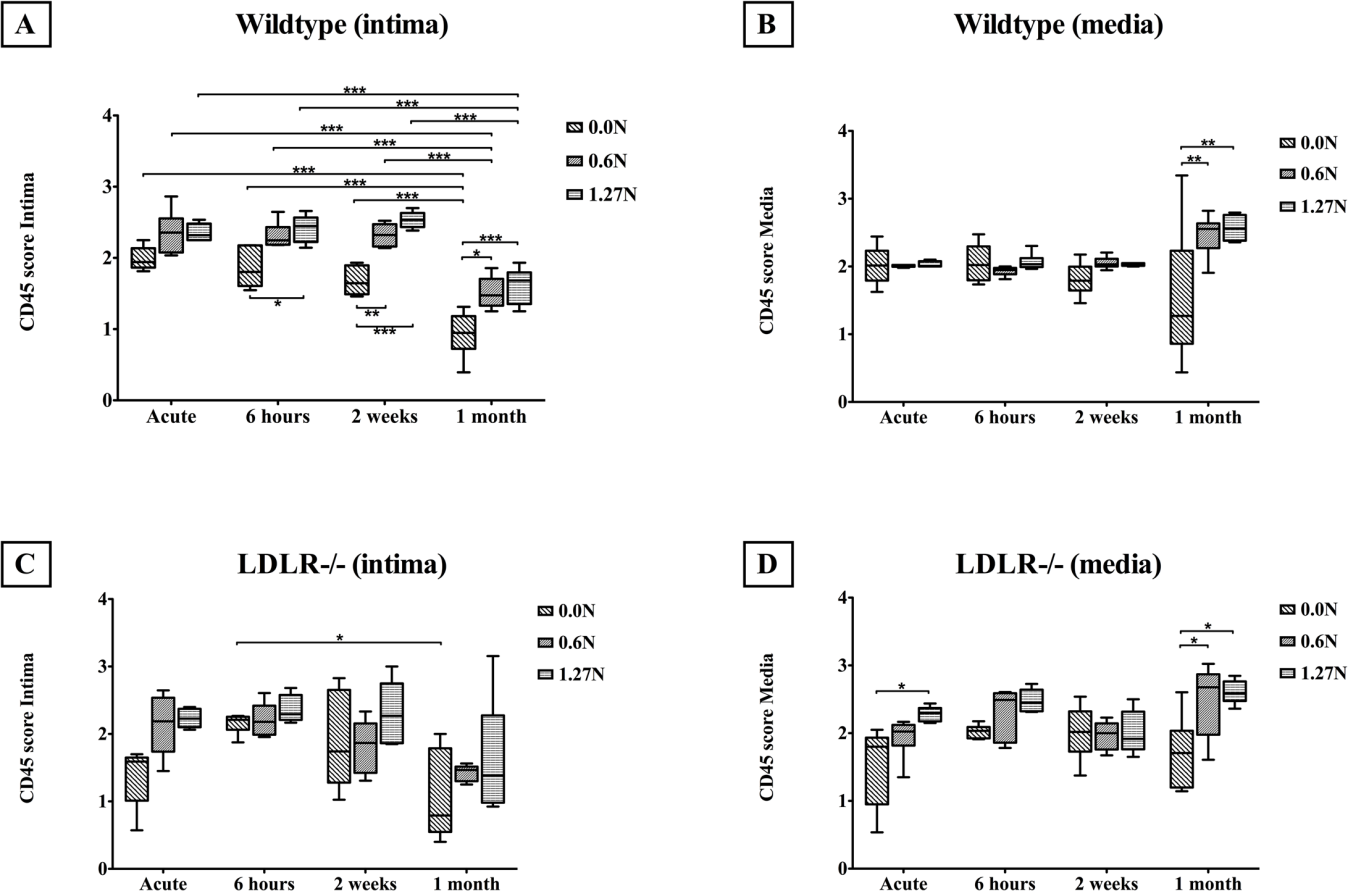
Inflammation after clamping in the intima (A,C) and media (B,D) in wildtype (A,B) and *LDLR-/-* (C,D) arteries (Median and IQR, 5–95 percentile). * p<0.05, ** p<0.001, *** p<0.0001.

CD45 staining allowed investigation of inflammation in the tunica intima and tunica media (Fig 8).

#### Tunica intima

Intimal CD45 score in wildtype (p<0.0001) as well as atherosclerotic (p = 0.0021) vessels is elevated after clamping but diminishes through time in both genotypes (p<0.0001, Fig 8A and 8C). While the intima in wildtype arteries shows higher CD45 content compared to *LDLR-/-* vessels in the acute condition (p = 0.0047), no difference is observed after 6 hours (p = 0.5175), 2 weeks (p = 0.2015) and 1 month (p = 0.9013). CD45 score is higher for wildtype segments compared to *LDLR-/-* samples after clamping with 0.6N (p = 0.0070), while no difference is seen between genotypes in the unclamped (p = 0.9969) and 1.27N (p = 0.4020) groups.

#### Tunica media

Medial CD45 in wildtype (p = 0.0052) and atherosclerotic arteries (p<0.0001) was elevated after clamping (Fig 8B and 8D). Through time, CD45 levels of unclamped arteries remain stable (p = 0.2557), while CD45 score in the tunica media raises in 0.6N (p = 0.0004) and 1.27N (p<0.0001) clamped vessels. Medial CD45 levels in wildtypes remain stable (p = 0.2304, Fig 8B), but CD45 content increases through time in atherosclerotic vessels (p = 0.0064, Fig 8D). Medial CD45 abundance is higher in *LDLR-/-* than wildtype segments in the 1.27N condition (p = 0.0010), but does not differ between genotypes in unclamped (p = 0.8139) and 0.6N clamped (p = 0.4720) segments. After 6 hours, atherosclerotic arteries display a higher CD45 content in the tunica media compared to wildtype vessels (p = 0.0016), while no difference is observed between genotypes in the acute (p = 0.3029), 2 weeks (p = 0.6996) and 1 month (p = 0.6794) conditions.

## 4. Discussion

In this chapter we have described that arteries of wildtype and atherosclerotic mice show a different time-dependent response to *in vivo* clamping. Endothelial cell presence and function, SMC phenotype and function, the elastic membrane curvature and inflammation were investigated.

### 4.1. Endothelium

We observed an immediate drop in endothelium-dependent vasodilatation in wildtypes after clamping. Remarkably, arteries of *LDLR* knockout mice did not show deterioration of endothelial function after clamping. This might be explained by the trend toward lower baseline relaxation in unclamped *LDLR-/-* compared to wildtype samples. Apparently, this reduces additional deterioration of endothelial function by clamping. Immunohistochemical staining of CD105 showed endothelial loss following clamping, confirming the vasoresponse tests. Endoglin or CD105 is a type I integral membrane glycoprotein located on cell surfaces. It is expressed at low levels in resting endothelial cells, but is highly expressed in vascular endothelial cells at sites of active angiogenesis such as tumor vessels, inflamed tissues, healing wounds and upon vascular injury [16]. While clamping-induced loss of endothelial function was recovered after one month in wildtype arteries, CD105 level was not. Since we also observed a drop in CD105 score through time in unclamped arteries, it seems that even freeing an artery of surrounding fat results in endothelial loss on the long term. This might be an explanation for the absence of a statistical effect of clamping in the chronic time points. The somewhat lower baseline endothelial function after 1 month might be caused by slow deterioration of endothelial function with aging [13].

### 4.2. Innermost elastic membrane

The wavy pattern of medial elastic membranes of large arteries serves to enable expansion of the vessel lumen to damp the pulse waves originating from the systole, which provides energy to elastically recoil the wall during diastole (Windkessel effect). In unclamped segments, we observed a less wavy pattern of the innermost elastic membrane in *LDLR* knockout vessels compared to wildtypes samples. Furthermore, we have shown that clamping results in elastica flattening in wildtype arteries, and to a higher extent in atherosclerotic arteries. This acute effect of arterial clamping confirms previous studies that also demonstrated flattening of medial elastic membranes following *ex vivo* [17] and *in vivo* [3] clamping. An interesting finding is that the flattening of the innermost elastic membrane in wildtype arteries after clamping is reversible in time and fully recovers within 1 month post-clamping. In contrast, arteries in atherosclerotic mice show no recovery. Local disruption of the wavy elastic membrane pattern could cause incongruence in the elastic properties of the clamped vessel, resulting in the initiation of standing waves and a change in diastolic/systolic difference. Hence, clamping load should be limited especially when clamping atherosclerotic arteries, because they are more susceptible to elastica flattening which does not recover in the same time span as healthy vessels.

### 4.3. Smooth muscle cells

Medial contractile SMC react to vascular injury such as arterial clamping by dedifferentiating into a synthetic, mobile phenotype [10]. While α-SMA is highly present in contractile SMC, the synthetic phenotype is characterized by osteopontin. As expected, immunohistochemical staining for those markers demonstrated higher baseline osteopontin content in unclamped arteries of mice with atherosclerosis compared to wildtype vessels. This can be explained by the hyperlipidemic serum profile in *LDLR* knockout mice, which has been shown to upregulate osteopontin expression [18]. Jackiewicz et al. described a phenotypic switch to synthetic SMC (transmission electron microscopy) 7 days after *in vivo* clamping of rat arteries, which was reversed at 14 days [10]. We observed a rise in osteopontin 6 hours and 2 weeks after clamping, which was reversed after 1 month. This difference in time course could be explained by the difference in techniques used for analysis, the type of clamp or the animal model. We observed a global rise in osteopontin content through time, for wildtype as well as knockout arteries. This effect was detected not only in the clamped segments, but also in unclamped samples. Therefore we suggest that osteopontin could be upregulated during the aging process, or that manipulation to dissect the vessel from surrounding tissue is a trigger to induce a phenotypic switch. SMC function was studied by SNP, either immediately after clamping or 1 month post-op. We did not detect a change in functional integrity of SMC at these time points, which confirms our histological findings.

### 4.4. Inflammation

Our results show that clamping results in a higher CD45 content in wildtype as well as atherosclerotic arteries, both in the tunica media and tunica intima. This is consistent with studies investigating the consequences of mechanical injury to arteries [19,20]. While inflammation in the intima diminishes through time, medial CD45 score remains elevated up to one month after clamping. This could be explained by an initial adherence of inflammatory cells to the damaged endothelium, with subsequent migration into the deeper arterial wall layers, since it was demonstrated that pathology could influence the permeability of the innermost elastic membrane [21]. Moreover, as has been described, inflammatory cells can also enter the media via the vasa vasorum in the tunica adventitia in response to injury [22,23]. Clamping at 1.27N results in higher medial CD45 content in arteries of atherosclerotic mice compared to healthy vessels, while a load of 0.6N leads to lower intimal CD45 score in knockout versus wildtype samples. Also, we observed that intimal inflammation in wildtypes is higher compared to *LDLR-/-* segments in the acute condition. However, medial inflammation is lower in arteries of wildtype versus knockout mice 6 hours after clamping. This suggests that inflammation after clamping in diseased arteries is most prominent in the media, and for healthy arteries in the intima. This might also be explained by a higher elastica permeability in vessels of atherosclerotic mice [21].

## 5. Conclusions and Future Perspectives

We have demonstrated that clamping up to 1.27N, which is approximately four times the minimal occlusion force [3], induces damage to mouse thoracic aortas that is not fully repaired after one month. Interestingly, the most pronounced effect was observed on the level of the innermost elastic membrane and not at the endothelium. Also, inflammation in the tunica media appears to be of higher importance than inflammation in the intima.

An interesting elaboration of our current study would be to investigate the vessel wall response to injury at a molecular level. Given the important roles of oxidative stress and metalloproteinases in vascular remodeling and stress response, these components should be studied to comprehend the full nature of the observed wall reaction. Also, endothelial cell activation could be studied by markers like vascular cellular adhesion molecule 1 or endothelial microparticles.

Since even these low clamping forces induce vascular damage, it is clear that forces during arterial clamping (intentional or unintentional) should be kept to an absolute minimum. To this purpose, safety features or limits could be placed on the surgical instruments in future robot-assisted surgical systems. However, the tolerable limit, as was shown in this study, will differ for atherosclerotic tissue as compared to healthy tissue. Therefore, pathology or even patient-specificity should be taken into account when considering these kinds of safety limits.

One way to incorporate this pathology- or patient-specificity is through finite element simulation, in which a computational model of the tissue is made to represent its mechanical response to mechanical loading, as shown for arterial clamping by our group in [24]. This model can be incorporated into the control of the robotic surgical device, upon which patient-specific limits can be calculated and maintained in real-time during the surgical procedure.

A key element in these models is the description of a material model that can accurately describe the mechanical behavior of the tissue as it is being damaged, and to tune the parameters of this model to a certain tissue type and pathology. The results obtained in the current study can serve as the input for this tuning process, analogous to the approach described in [25] for rat abdominal arteries, but now incorporating the essential aspect of repair time.

To start the translational track to human medicine, analogous experiments to those performed here will have to be performed in large animal *in vivo* experiments and *ex vivo* human arteries. In [26], we have for example already quantified the difference in mechanical behavior of healthy and pathological human aortic tissue. Our final aim is to transfer this concept to the operating theater, where it could be implemented in surgical robots to improve patient safety during robotic minimally invasive procedures.
